# Association of Maternal Diet with Infant Birthweight in Women with Gestational Diabetes Mellitus

**DOI:** 10.3390/nu15214545

**Published:** 2023-10-26

**Authors:** Aikaterini Apostolopoulou, Antigoni Tranidou, Violeta Chroni, Ioannis Tsakiridis, Emmanuella Magriplis, Themistoklis Dagklis, Michail Chourdakis

**Affiliations:** 1 Laboratory of Hygiene, Social & Preventive Medicine and Medical Statistics, School of Medicine, Faculty of Health Sciences, Aristotle University of Thessaloniki, 541 24 Thessaloniki, Greece; katapost@yahoo.gr (A.A.); andie.t.1989@gmail.com (A.T.); violetaxroni@gmail.com (V.C.); mhourd@gapps.auth.gr (M.C.); 2 3rd Department of Obstetrics and Gynecology, School of Medicine, Faculty of Health Sciences, Aristotle University of Thessaloniki, 541 24 Thessaloniki, Greece; themistoklis.dagklis@gmail.com; 3 Department of Food Science and Human Nutrition, Agricultural University of Athens, Iera odos 75, 118 55 Athens, Greece; emagriplis@aua.gr

**Keywords:** nutrition, pregnancy, macronutrients, micronutrients, diet, SGA, GDM

## Abstract

Objective: This study aimed to explore the potential impact of pre-pregnancy and early pregnancy maternal nutrition on the incidence of small-for-gestational-age neonates (SGA) in women with gestational diabetes mellitus (GDM). Methods: A prospective cohort study was conducted between 2020 and 2022 at the 3rd Department of Obstetrics and Gynaecology (School of Medicine, Faculty of Health Sciences, Aristotle University of Thessaloniki, Greece). Pregnant women from routine care were surveyed about their dietary habits during two distinct periods: six months prior to pregnancy (period A) and from the onset of pregnancy until the oral glucose tolerance test at 24–28 gestational weeks (period B). The intake of various micronutrients and macronutrients was quantified from the questionnaire responses. Logistic regression models, adjusted for potential confounders including age, pre-pregnancy body mass index (BMI), smoking status, physical activity and parity, were used to evaluate the association between nutrient intake and small-for-gestational-age neonate incidence. Results: In total, 850 women were screened and of these, 90 (11%) were diagnosed with gestational diabetes mellitus and were included in the study. There were significant associations between the intake of specific nutrients and the occurrence of small-for-gestational-age neonates; higher fat intake compared to non-small for gestationa age during period B (aOR: 1.1, *p* = 0.005) was associated with an increased risk for small-for-gestational-age neonates, while lower intake of carbohydrates (g) (aOR: 0.95, *p* = 0.005), fiber intake (aOR: 0.79, *p* = 0.045), magnesium (aOR: 0.96, *p* = 0.019), and copper (aOR:0.01, *p* = 0.018) intake during period B were significantly associated with a decreased risk for small-for-gestational-age neonates. Conclusions: The findings of this study highlight the potential role of maternal nutrition in modulating the risk of small for gestational age neonatesamong women with gestational diabetes mellitus. The results advocate for further research on the assessment and modification of both pre-pregnancy and early pregnancy nutrition for women, especially those at higher risk of gestational diabetes mellitus, to reduce the risk of gestational diabetes mellitus.

## 1. Introduction

Fetal growth restriction (FGR) and small-for-gestational-age neonates (SGA) confer an increased risk of perinatal morbidity and mortality [1], while the incidence of adverse outcomes is largely dependent on the severity of growth restriction [1]. Small for gestational age (SGA) is defined as a birth weight less than the 10th percentile for gestational age. Moreover, SGA neonates born to women with gestational diabetes mellitus (GDM) are at higher risk for adverse neonatal outcomes when compared to those born with adequate weight or large for gestational age (LGA) [2]. The presence of GDM may disrupt the normal metabolic processes that occur during pregnancy by affecting fetal growth and development [3]. The altered maternal glucose metabolism, along with potential vascular complications, may pose an obstacle to the transfer of nutrients to the developing fetus, leading to impaired fetal growth and an increased likelihood of SGA [4]. In addition, SGA is an independent risk factor for adverse neonatal outcomes and mortality in women with GDM, irrespective of maternal age, weight gain, glucose levels, and gestational conditions; neonatal complications experienced in SGA include hypoglycemia, hypocalcemia, hyperbilirubinemia, and polycythemia [5]. SGA has been also linked to considerably higher odds of adverse outcomes in women with GDM, including respiratory distress syndrome (RDS), neonatal demise (NND), stillbirth, and jaundice [6].

A salient pathophysiological link between SGA and GDM is insulin resistance. In GDM-affected pregnancies, a compromised ability to efficiently use insulin can result not only in macrosomia but also in the SGA phenotype under distinct conditions. The placenta’s function in this dynamic is noteworthy. Instances of placental insufficiency, characterized by diminished nutrient and oxygen transfer capabilities, serve as a significant contributor to the emergence of SGA neonates. In the context of GDM, the balance of nutrient supply might be disrupted either through excess or deficiency, thereby modulating fetal growth patterns [4].

The role of inflammation cannot be understated. Chronic inflammation, a hallmark in GDM, is concomitantly observed in SGA neonate presentations. This suggests a potentially adversarial role of inflammatory pathways in fostering optimal fetal growth. Maternal vascular health, especially endothelial functionality, is another critical determinant. Evident dysfunction, present in both GDM and pregnancies predisposed to SGA, may alter placental perfusion patterns, adding complexity to the growth outcomes [7].

Furthermore, oxidative stress, a recurrent element in many pathological states, is also implicated. This phenomenon can be detrimental to both maternal and fetal tissues, thus influencing the spectrum of pregnancy outcomes and increasing the risk of SGA. Overall, these intertwined pathophysiological pathways accentuate the intricacy inherent in pregnancies affected by GDM and the consequential fetal growth patterns, underscoring the need for comprehensive research and vigilant clinical approaches [8].

Furthermore, it’s important to note that SGA represents a distinct risk factor for unfavorable neonatal outcomes and mortality in women with GDM, regardless of factors such as maternal age, weight gain, glucose levels, and gestational circumstances. Neonatal complications commonly observed in SGA infants encompass hypoglycemia, hypocalcemia, hyperbilirubinemia, and polycythemia [5]. 

SGA has also been associated with significantly increased chances of adverse outcomes in women with GDM, which encompass conditions like respiratory distress syndrome (RDS), neonatal demise (NND), stillbirth, and jaundice [6].

Dietary intake is a cornerstone in the management of GDM. A well-balanced and adequate diet is essential not only for maintaining maternal blood glucose levels within a normal range but also for ensuring optimal fetal growth and development. Carbohydrates, being the primary dietary component influencing postprandial blood glucose levels, are the central focus in GDM nutritional management. The type (complex vs. simple), amount, and distribution of carbohydrates throughout the day can impact maternal glucose control. For instance, diets rich in high-fiber and low-glycemic-index foods have been suggested to help manage postprandial glucose levels better in pregnant women with GDM [9].

Interestingly, the nutritional strategies that aim to regulate blood glucose in GDM could also have implications for fetal growth. When maternal glucose levels are not adequately controlled, there is an increased risk for macrosomia or having a baby with a higher birth weight. However, if dietary restrictions are too stringent, or if caloric intake is insufficient, there might be a risk of the fetus not receiving enough nutrients, potentially leading to SGA [10].

A study conducted in Dhaka, Bangladesh, from April 2011 to June 2012 [11] assessed maternal characteristics and nutritional status in early pregnancy and their impact on neonatal birth weight. The study found multiple maternal micronutrient deficiencies in early pregnancy, including vitamin D, vitamin B12, and iron. Interestingly, while GDM prevalence was higher in overweight women, maternal BMI in early pregnancy was not related to preterm deliveries or low birth weight (LBW). However, LBW was associated with lower folate levels, elevated cord triglyceride concentrations in neonates, the mother’s height, and increased maternal homocysteine levels. These findings underscore the intricate relationship between maternal nutrition, GDM, and fetal growth outcomes, especially in the context of SGA.

Additionally, broader dietary habits can modulate inflammatory responses and oxidative stress, both of which have been linked to adverse pregnancy outcomes, including SGA. As evidence continues to increase, it becomes clear that promoting comprehensive dietary patterns, rather than isolated nutrient supplementation, might be the key to optimizing maternal and neonatal health outcomes, especially in pregnancies complicated by GDM.

In essence, the nutritional management of GDM is a delicate balance. While the primary objective is to maintain maternal blood glucose within a target range, care must be taken to ensure that dietary recommendations do not inadvertently lead to restricted fetal growth. Continuous monitoring, personalized diet plans, and a comprehensive understanding of nutritional needs during pregnancy are crucial to prevent complications like SGA in pregnancies complicated by GDM.

The effect of nutrient intake on fetal growth has been extensively studied; existing evidence concluded that preconceptual supplementation with folic acid significantly reduces the risk of SGA at birth independent of other risk factors [12]. There are also indications of increased risk in SGA births from high caffeine intake, particularly over 300 mg of caffeine per day [13]. Lipid-based nutrient supplements have a slight, positive effect on SGA and newborn stunting compared to iron and folic acid supplements [14]. The effect of certain dietary patterns on SGA incidence has been examined in a randomized trial from Spain, in which adherence to a Mediterranean diet was compared with usual care and was shown to significantly reduce the incidence of SGA [15].

The prevalence of SGA has been associated with lower BMI before pregnancy and lower weight gain during pregnancy [12]. Balanced energy/protein supplementation may decrease the risk for SGA, while another study suggested that the risk of SGA increased significantly following high protein supplementation (≥25% energy from protein) [13]. Low glycaemic index diets were also associated with a twofold increased risk of SGA [16]. Furthermore, maternal vitamin D deficiency and iron deficiency, with or without anemia, have been associated with a higher risk of SGA [17,18,19].

The present study aimed to evaluate the impact of the nutritional habits of Greek women prior to pregnancy and during pregnancy prior to the diagnosis of GDM on the risk of SGA.

## 2. Methods

### 2.1. Study Participants

This is a sub-group analysis of a prospective epidemiological study named BORN 2020, conducted on Greek pregnant women between December 2020 and October 2022. All participants were recruited from routine care at the scheduled appointment for the first trimester scan (11^+0^–13^+6^ gestational weeks) at the 3rd Department of Obstetrics and Gynecology (School of Medicine, Faculty of Health Sciences, Aristotle University of Thessaloniki, Greece). The sample was derived from pregnant women proceeding with routine care. Sample randomization was achieved with the use of random numbers tables. The selection criteria used to determine eligibility were pregnancy, age >18 years, use of Greek language, and the diagnosis of GDM. Women were excluded if they were on a specific nutritional program due to any health issue (e.g., malabsorption syndrome, inflammatory bowel disease, etc.) or were diagnosed with certain preexisting medical conditions, including pre-existing diabetes, chronic hypertension, and autoimmune diseases. Participants were surveyed about their dietary habits during two distinct periods: six months prior to pregnancy (period A) and from the onset of pregnancy until the oral glucose tolerance test at 24–28 gestational weeks (period B). Sociodemographic data, medical history, maternal age, maternal weight, data on smoking habits during pregnancy, chronic conditions, medications, parity and gravidity, and method of conception were collected. The primary outcome of interest was SGA, defined as neonates born less than 10th centile.

The study design was approved by the Bioethics Committee of the Aristotle University of Thessaloniki, Greece (6.231/29 July 2020). After obtaining written informed consent, a planned interview during the first and second visits followed to collect all data.

### 2.2. Dietary Assessment

In order to record the dietary habits of the participants in Supplementary Materials, a questionnaire recently developed by our team was used, which has been previously validated in the Greek pregnant population [20]. This is a semi-quantitative questionnaire based on two pre-existing food consumption questionnaires for the nutritional assessment of Mediterranean populations [21,22]. The participants were asked to fill in the questionnaire, recording the frequency of consumption of each food; available options of response included: “*never*”, and “*x portions daily/per week/per month*”. Frequency reported per food group response was converted into daily intake. Frequency per day consumed was then quantified by multiplying with portion size reported. Finally, grams consumed were converted into nutritional data and micronutrient intakes were calculated. The procedure included personal interview for all the participants. Data during the first visit included nutritional habits 6 months prior to conception and nutritional habits during pregnancy.

### 2.3. Statistical Analysis

Frequencies and descriptive statistics were expressed as n (%) and mean (± standard deviation (SD)) for parametric data, or median (25th–75th interquartile range (IQR)) for non-parametric data, respectively. Sample size estimation was based on relevant studies with 80% power and 95% confidence intervals. Before hypothesis testing, data were examined for normality using Shapiro–Wilk test. Multiple logistic regression analysis was performed for evaluating risk of SGA based on nutrient intake, adjusted for possible confounders: weight, maternal age, smoking, parity, and method of conception (spontaneous/assisted reproductive technology (ART)). Data were analyzed using the programming language R.

## 3. Results

The study cohort consisted of 850 women. Of these, 90 women (11%) were diagnosed with GDM, as can be seen in Table 1. The mean age of the included patients was 34.3 years, and the median pre-pregnancy BMI was 23.5. As shown in Table 2, none of the maternal characteristics of the study population were significantly associated with an increased risk for SGA.

In terms of nutrient intake, our analysis revealed several significant associations with the risk of SGA. Notably, water intake during both periods was associated with a statistically significant but small decrease in SGA risk (aOR: 0.99, 95% CI: 0.99–0.99, *p* = 0.034 for period A; aOR: 0.99, 95% CI: 0.99–0.99, *p* = 0.014 for period B). Carbohydrate intake during period B was associated with a 5% lower risk (aOR: 0.95, 95% CI: 0.91–0.98, *p* = 0.005), fiber intake during period B was also associated with 21% reduced risk for SGA (aOR: 0.79, 95% CI: 0.6–0.96, *p* = 0.045), while fat intake during the same period increased the risk by 10% (aOR: 1.1, 95% CI: 1.03–1.2, *p* = 0.005). Added sugar intake during period B seemed to lower the risk for SGA by 4% (aOR: 0.96, 95% CI: 0.92–0.99). All significant associations with nutrient intake are presented in Table 3.

Table 4 provides an overview of the association between the intake of certain food items and SGA. Fresh salad intake during period B (*p* = 0.039), legume intake during period A (*p* = 0.031), and sunflower seed oil intake during period B (*p* = 0.038) were all significantly associated with SGA.

Moreover, Table 5 presents the results of the analysis on the association of the preferred cooking methods with SGA. When the preferred method of cooking during gestation was “fried”, the association with SGA was increased (*p* = 0.015).

## 4. Discussion

### 4.1. Main Findings

The results of the present study suggest that certain dietary habits and nutrient intakes can influence the risk for SGA in women diagnosed with GDM. More specifically, (1) carbohydrate intake during pregnancy lowered the risk of SGA by 5%, (2) fat intake during pregnancy increased the risk of SGA by 10%, (3) fiber intake during pregnancy was associated with a 21% reduced risk of SGA, (4) magnesium intake during pregnancy was found to decrease the risk of SGA by 4%, (5) copper intake during pregnancy reduced the SGA risk by 99%, (6) frying food was associated with an increased risk of SGA in women with GDM, (7) less water intake before and during pregnancy was associated with a small decrease in SGA risk. Specifically, for each gram of water consumed, the risk of SGA decreased by 0.1%.

### 4.2. Interpretation of Findings

Maternal age seemed to be a risk factor for SGA in another study [23]. Moreover, a recent study indicated that overweight and obesity before pregnancy, depending on gestational weight gain, were associated with an increased risk for SGA in GDM individuals [24]. In the study [25] conducted by Sweeting et al., they suggested that a higher-than-recommended intake of carbohydrates during pregnancy supports normal fetal growth. This aligns with our findings, where we observed that a higher carbohydrate intake during pregnancy (83% of total energy intake) lowered the risk of SGA by 17%. However, it is important to note that the specific types and sources of carbohydrates may also play a significant role in these outcomes. Another study did not support our result that carbohydrate consumption before pregnancy may slightly decrease the SGA possibility, as no significant association was revealed [26]. In addition, while our findings indicate a promising association between maternal higher fiber intake and reduced SGA risk, more research is needed to validate and expand upon these results. Moreover, a study examining copper concentrations in pregnancy with SGA risk did not result in a significant association [27].

It has been documented that intracellular magnesium levels are lower in both children with diabetes mellitus and those who are obese [28]. In groups with type 2 diabetes mellitus and obesity, platelets exhibited a robust response to insulin. In states of insulin resistance, magnesium levels decrease prior to the onset of reduced insulin reactivity in platelets. Based on these findings, a decrease in magnesium levels occurs earlier than the development of poor insulin reactivity in platelets exposed to an insulin-resistant environment. This implies that low magnesium levels may be an inherent anomaly in infants with low birth weight. A study from Japan demonstrated that the average basal magnesium, but not plasma magnesium, is lower in SGA compared to appropriate-for-gestational-age (AGA) groups [29]. Basal magnesium levels were found to significantly correlate with birth weight as well as cord plasma leptin and IGF-1. Magnesium, as well as leptin and IGF-1, reflect the extent of fetal growth. Moreover, magnesium significantly correlates with the quantitative insulin sensitivity check index (QUICKI). The reduced magnesium in SGA may underlie the initial pathophysiological events that lead to insulin resistance.

A systematic review [30] examining the possible association of copper levels in the umbilical cord blood and birth weight showed that copper levels in the umbilical cord blood in SGA neonates were higher than copper levels in AGA. Throughout pregnancy, there is a noteworthy rise in plasma copper levels, which tend to return to their typical non-pregnant levels after childbirth. This increase may be linked, at least in part, to the production of ceruloplasmin, a major copper-binding protein influenced by fluctuations in estrogen levels (as observed by Izquierdo Alvarez et al. in 2007) [31]. While lower copper levels have been noted in the placentas of pregnancies resulting in SGA infants, there is a lack of comprehensive data regarding maternal plasma copper concentrations concerning SGA pregnancies.

Lipid peroxidation is closely associated with oxidative stress, a condition where there is an imbalance between substances that promote oxidation (pro-oxidants) and those that counteract it (antioxidants). This imbalance results in the excessive generation of reactive oxygen species (ROS) [32]. Such oxidative stress-induced damage is a significant factor in adverse pregnancy outcomes, including fetal growth restriction leading to SGA births. While an increase in oxidative stress during pregnancy can be considered a normal physiological occurrence in regular births, there are findings that indicate an abnormal intensification of this process in umbilical cord samples from SGA infants. This intensified oxidative stress is linked to the upregulation of various prostaglandins (PGs), eicosanoids, and oxygenated polyunsaturated fatty acids (oxy-PUFAs). Notably, an excess of PGs and thromboxane has been associated with fetal growth retardation [33]. On the contrary, a stable breakdown product of PGI2 or prostacyclin known as 6-keto-PGF1α, which serves as a valuable marker for PGI2 in humans, was found to be lower in large-for-gestational-age (LGA) newborns compared to SGA and appropriate-for-gestational-age (AGA) infants [34]. Given that PGI2 is a molecule that promotes fat storage, the reduced levels of its precursor in LGA infants may indicate a downregulation, suggesting sufficient subcutaneous adipose tissue. Moreover, the levels of this marker were inversely related to birth anthropometric measurements, with most of these associations persisting at 4 months of age. They also exhibited a positive correlation with the homeostatic model assessment of insulin resistance (HOMA-IR) levels, suggesting that the precursor of PGI2 might play a role in the fat accumulation process during catch-up growth and subsequent changes in insulin sensitivity. Additionally, the metabolite 12,20-DiHETE, derived from arachidonic acid, was differentially regulated in SGA, LGA, and AGA newborns. Lipids stemming from arachidonic acids have been implicated in the development of complications related to obesity, including diabetes and insulin resistance [35,36]. In a recent study [37], cord blood levels of 12,20-DiHETE were low in LGA newborns and high in SGA infants compared to AGA newborns. These levels are inversely correlated with birth weight and positively correlated with HOMA-IR levels at 4 months of age. This suggests that the higher levels observed in SGA infants may contribute to the development of lipotoxicity following excessive catch-up growth in weight.

Although available evidence does not focus on the correlation between micronutrient intake and incidence of SGA, recent data on preconceptional and periconceptional micronutrient supplementation show a possible effect on SGA [38]. In particular, a risk reduction of SGA was observed by mineral and vitamin supplementation according to a systematic review 26. In a meta-analysis, it was shown that multiple micronutrient supplementation is associated with reduced SGA risk compared to iron-only supplements [39]. There is a lack of explanations regarding potential mechanisms for the association of SGA in GDM with certain nutrients among the current data. Further investigations are warranted to elucidate this matter.

Moreover, regarding maternal nutrition, certain micronutrients play pivotal roles in ensuring optimal fetal growth and development, especially in the context of gestational diabetes mellitus (GDM). Calcium, an integral component of the skeleton, is vital for the development of the fetal skeletal system. Iron, essential for oxygen transport and energy metabolism, ensures that the fetus receives adequate oxygen, with deficiencies potentially leading to iron deficiency anemia in the mother and impacting fetal growth. Magnesium and phosphorus are involved in various metabolic processes, supporting fetal energy production and the development of bones and teeth, respectively. Potassium’s role in establishing membrane potential is crucial for the electrical activity in fetal nerve fibers and muscle cells. Folate, indispensable for DNA replication, is paramount for cell division in the rapidly growing fetus. A deficiency can lead to megaloblastic anemia and has been linked to adverse health effects, potentially increasing the risk of SGA. Vitamins A, B6, C, and E each have unique roles, from supporting vision and cell integrity to aiding in amino acid metabolism and providing antioxidant activity. A deficiency in any of these vitamins can lead to a range of issues, from xerophthalmia due to Vitamin A deficiency to neurological abnormalities from a lack of Vitamin B6. In the context of GDM, where maternal glucose regulation is paramount, these micronutrients can influence both maternal health and fetal growth patterns. For instance, imbalances or deficiencies, especially when combined with the metabolic disturbances of GDM, might contribute to an increased risk of SGA. Thus, while managing GDM, it is imperative to ensure that the mother’s diet is not only balanced in macronutrients but also adequately fortified with these essential micronutrients to safeguard against potential complications like SGA [40,41,42,43,44,45,46,47,48,49,50,51,52].

Beyond the specific nutrients and dietary habits explored in this study, broader dietary patterns, such as adherence to a Mediterranean diet (MD) pattern, have been shown to influence pregnancy outcomes; a recent Greek cohort study highlighted that women who adhered to a MD prior to conception exhibited a reduced risk of developing GDM [53]. This is particularly relevant given that GDM is associated with the risk of SGA neonates. Furthermore, international guidelines on antenatal nutrition emphasize the importance of specific nutritional components, i.e., folic acid and iron supplementation, during pregnancy [54]. These considerations highlight the importance of comprehensive dietary patterns to improve pregnancy outcomes.

This study has strengths and limitations. To our knowledge, this is the first study assessing in a Greek population the impact of nutrition in women with GDM on SGA. Moreover, we managed to examine all possible risk factors for SGA, including the demographic and nutritional ones. The methodology used was proper and thorough and the questionnaire used was a validated short tool developed to fulfill the need for quick dietary assessment. However, there are several limitations. The sample size was not large and therefore any generalization of the results needs to be done with caution. Data regarding preconceptional nutrition were obtained retrospectively, so recall bias may have occurred.

The present study has tried to shed light on various aspects of SGA etiology. It highlighted the potential influence of specific dietary factors on the risk of SGA in women with GDM. The identification of maternal dietary factors associated with SGA outcomes during distinct pregnancy periods provides valuable insights for guiding nutritional interventions and prenatal care. Targeted dietary counseling aimed at optimizing nutrient intake, especially the consumption of nutrient-dense foods, may offer promising strategies to improve pregnancy outcomes for women with GDM and reduce the risk of SGA. However, existing evidence is not robust and safe enough to justify innovative interventions. More research is needed to further enhance our understanding and pave the way for optimal pregnancy outcomes by implementing effective treatment and prevention measures to mitigate the occurrence of adverse effects.

## Figures and Tables

**Table 1:** Maternal characteristics of study population (*n* = 90).

Maternal Characteristics	*n* (%) or Mean ± SD or Median (IQR)
Age	34.3 ± 4.4
Age > 35	39 (43.3)
Weight before pregnancy (kg)	64.5 (58.3–76.5)
BMI before pregnancy (kg/m^2^)	23.5 (21.4–27.9)
BMI category (before pregnancy)	
Overweight	33 (36.7)
Obese	15 (16.7)
Weight 1st trimester (kg)	65 (59–75)
Parity 0 1 2	43 (47.8) 37 (41.1) 10 (11.1)
Smoking during pregnancy	14 (15.6)
Scheduled pregnancy	78 (86.7)
ART	7 (7.8)
SGA Birth weight (g) 3208.3 ± 499.1	11 (11.2)
Birth weight (g)	3227 (2970–3508)

Data are presented in mean ± SD or *n* (%). BMI: body mass index; ART: assisted reproductive technology.

**Table 2:** Maternal characteristics separately for SGA vs. non-SGA women with GDM.

General Maternal Characteristics of GDM Women	SGA (N = 11)	Non-SGA (N = 79)	*p* Value
Weight before pregnancy	63 (60.8)	65 (58.7)	0.39
BMI before pregnancy	23.4 (22.0, 31.4)	23.5 (21.3, 27.1)	0.28
Age	33.5 (±4.2)	34.4 (±4.5)	0.53
GA at birth	12.43 (12.36, 12.57)	12.57 (12.3, 12.8)	0.19
Weight 1st trimester	63 (60.8)	65 (58.7)	0.39
ART	0 (0)	7 (8.9)	-
Smoking	1 (9.1)	13 (16.5)	0.85
BMI >25	4 (36.4)	29 (36.7)	0.76
BMI >30	3 (27.3)	12 (15.2)	0.56
Age >35	4 (36.4%)	35 (44.3%)	0.86
FGR	1 (9.1%)	1 (1.3%)	0.58

BMI: body mass index; ART: assisted reproductive technology; FGR: fetal growth restriction; GA: gestational age; SGA: small for gestational age; *t*-test or Mann–Whitney test depending on data distribution. Level of significance *p* ≤ 0.05.

**Table 3:** Macronutrient and micronutrient intake for the outcome SGA.

Nutrients	SGA	
	aOR (95% CI)	*p* Value
Energy (kcal)—A	1 (0.99, 1)	0.64
Energy (kcal)—B	1 (0.99, 1)	0.42
Water—A	0.99 (0.99, 0.99)	0.034
Water—Β	0.99 (0.99, 0.99)	0.014
Proteins (g)—A	1.06 (0.99, 1.16)	0.083
Proteins (%)—A	1.29 (0.99, 1.73)	0.067
Proteins (g)—B	1 (0.93, 1.08)	0.83
Proteins (%)—B	1 (0.77, 1.3)	0.98
Carbs (g)—A	0.98 (0.96, 1.01)	0.32
Carbs (%)—A	0.96 (0.88, 1.03)	0.3
Carbs (g)—B	0.95 (0.91, 0.98)	0.005
Carbs (%)—B	0.83 (0.72, 0.92)	0.004
Fat (g)—A	1 (0.96, 1.05)	0.66
Fat (%)—A	1.01 (0.93, 1.09)	0.65
Fat (g)—B	1.1 (1.03, 1.2)	0.005
Fat (%)—B	1.19 (1.07, 1.35)	0.003
Fiber—A	0.81 (0.64, 0.99)	0.062
Fiber—Β	0.79 (0.6, 0.96)	0.045
Vitamin A—A	0.99 (0.99, 1)	0.12
Vitamin A—B	1 (0.99, 1)	0.85
Vitamin D—A	1.21 (0.55, 2.35)	0.58
Vitamin D—B	1.68 (0.74, 3.73)	0.18
Vitamin K—A	0.98 (0.96, 1)	0.2
Vitamin K—B	1 (0.98, 1.02)	0.44
Pantothenic acid—A	1.09 (0.44, 2.57)	0.84
Pantothenic acid—B	0.59 (0.17,1.67)	0.36
Folic acid—A	0.99 (0.97, 1.01)	0.74
Folic acid—B	0.98 (0.96, 1.01)	0.3
Vitamin C—A	0.97 (0.93, 0.99)	0.052
Vitamin C—B	0.97 (0.94, 1)	0.15
Sodium—A	0.99 (0.99, 1)	0.16
Sodium—B	0.99 (0.99, 1)	0.18
Potassium—A	0.99 (0.99, 1)	0.5
Potassium—Β	0.99 (0.99, 1)	0.16
Magnesium—A	0.99 (0.97, 1)	0.32
Magnesium—Β	0.96 (0.93, 0.99)	0.019
Phosphorus—A	1 (0.99, 1)	0.8
Phosphorus—B	0.99 (0.99, 1)	0.27
Copper—A	0.22 (0.02, 1.36)	0.13
Copper—Β	0.01 (0, 0.33)	0.018
Iodine—A	0.98 (0.95, 1.01)	0.28
Iodine—Β	0.97 (0.94, 1)	0.12
Vegetable proteins—A	0.95 (0.8, 1.13)	0.59
Vegetable proteins—B	0.92 (0.77,1.07)	0.36

Multiple logistic regression model. Adjusted OR for age, BMI, smoking, physical activity, and parity. ART: assisted reproductive technology; aOR: adjusted odds ratio; BMI: body mass index A: before pregnancy; Β: during pregnancy; Statistical significant values are presented in bold. Level of significance *p* ≤ 0.05.

**Table 4:** Food items from periods A and B for the outcome SGA.

	*p* Value for Outcome SGA
White bread—A	0.653
White bread—B	0.694
Wholegrain bread—A	0.239
Wholegrain bread—B	0.114
Pasta white—A	0.199
Pasta white—B	1
Barley—A	0.873
Barley—B	0.327
Rice—A	0.117
Rice—B	0.111
Cereal- A	0.114
Cereal—B	0.364
Potatoes—A	0.638
Potatoes—B	0.345
Fresh fruit—A	0.095
Fresh fruit—B	0.076
Dried fruit—A	0.463
Dried fruit—B	0.539
Fresh salad—A	0.059
Fresh salad—B	**0.039**
Boiled salad—A	0.323
Boiled salad—B	0.425
Eggs—A	0.671
Eggs—B	0.585
Legumes—A	0.031
Legumes—B	0.721
Nuts—A	0.894
Nuts—B	0.682
Fish and seafood—A	0.808
Fish and seafood—B	0.793
Red meat—A	0.324
Red meat—B	0.450
Processed meat—A	0.870
Processed meat—B	0.918
White meat—A	0.266
White meat—B	0.722
Full-fat dairy—A	0.910
Full-fat dairy—B	0.592
Low-fat dairy—A	0.764
Low-fat dairy—B	0.091
Olive oil—A	0.663
Olive oil—B	0.523
Olives—A	0.816
Olives—B	0.524
Sunflower seed oil—A	0.087
Sunflower seed oil—B	**0.038**
Honey—A	0.970
Honey—B	0.891
Beverages—A	0.425
Beverages—Β	0.846
Sugar added in beverages—A	0.920
Sugar added in beverages—B	0.733
Soft drinks no sugar—A	0.338
Soft drinks no sugar—B	0.747
Sweets—A	0.930
Sweets—B	0.876
Alcohol—A	0.112
Alcohol—Β	0.821
Decaffeinated coffee—A	0.711
Decaffeinated coffee—B	0.334
Coffee—A	0.377
Coffee—B	0.906
Water—A	0.085
Water—B	**0.018**
Fast food—A	0.180
Fast food—B	0.579
Salted snacks—A	0.530
Salted snacks—B	0.486
Pies—A	0.689
Pies—B	0.984
Puff pastry—A	0.780
Puff pastry—B	0.043
Tea—A	0.417
Tea—B	0.964
Herbs—A	0.425
Herbs—B	0.846
Fresh Juice—A	0.607
Fresh Juice—B	0.664
Heavy cream low fat—A	0.016
Heavy cream low fat—B	0.003
Heavy cream full fat—A	0.980
Heavy cream full fat—B	0.950
Butter—A	0.984
Butter—B	0.706
Margarine—A	0.298
Margarine—B	0.116

SGA: Small for gestational age; A: before pregnancy; B: during pregnancy. *t*-test or Mann–Whitney test depending on data distribution. Statistical significant values are presented in bold. Level of significance *p* ≤ 0.05. Statistically significant values are presented in bold.

**Table 5:** Cooking method and SGA.

Cooking Method	*p*-Value
Fried—A	0.057
Fried—B	0.015
Boiled—A	0.270
Boiled—B	0.250
Grilled—A	0.412
Grilled—B	0.290

SGA: Small for gestational age; A: before pregnancy; B: during pregnancy. *t*-test or Mann–Whitney test depending on data distribution.

## Data Availability

Data is unavailable due to privacy restrictions concerning patient confidentiality.

## Supplementary Materials

The following supporting information can be downloaded at: https://www.mdpi.com/article/10.3390/nu15214545/s1.
