# Supplementary material for: Association of Maternal Diet with Infant Birthweight in Women with Gestational Diabetes Mellitus
Source: Nutrients. 2023 Oct 26;15(21):4545. doi: 10.3390/nu15214545 (PMC10648020; doi:10.3390/nu15214545)
Supplement: Supplementary file 1 [file nutrients-15-04545-s001.zip › nutrients-2683603-supplementary.pdf]

Τμήμα Ιατρικής
Σχολή Επιστημών Υγείας
Αριστοτέλειο Πανεπιστήμιο Θεσσαλονίκης

Ερωτηματολόγιο συχνότητας κατανάλωσης τροφίμων στην κύηση

Ονοματεπώνυμο: Ημερ. Γέννησης: / /

ID: Τρίμηνο: 1 2 3 PP

Παρακαλείσθε να σημειώσετε με τη μέγιστη δυνατή ακρίβεια σε 1 μόνο κουτάκι κάθε γραμμής, αυτό που ταιριάζει περισσότερο στις συνήθειές σας κατά τους τελευταίους μήνες, ως προς τις ενδεικτικές μερίδες που αναφέρονται

1 κ.σ = μέγεθος αντίχειρα	1 φλ. τσαγιού = 1 μεγάλη γροθιά	1 μερίδα κρέας = παλάμη του χεριού

1. Πόσες μερίδες από τις παρακάτω κατηγορίες τροφίμων καταναλώνετε κατά μέσο όρο;		σημειώστε αυτό που ταιριάζει περισσότερο			
	**Επεξήγηση μερίδας**	*Ποτέ / Σχεδόν ποτέ*	*Μερίδες /ημέρα*	*Μερίδες /εβδομάδα*	*Μερίδες /μήνα*
**Ψωμί** α) Λευκό	1 φέτα (τοστ)				
β) Ολικής άλεσης					
**Πίτες** α) Σπιτικές ή με φύλλο	1 τεμάχιο (6x6 εκ)				
β) Σφολιάτα					
**Μακαρόνια** α) Λευκά	1 κεσεδάκι γιαουρτιού				
β) Ολικής άλεσης					
**Κριθαράκι**	1 κεσεδάκι γιαουρτιού				
**Πατάτες**	1 κεσεδάκι γιαουρτιού				
**Ρύζι**	0,5 κεσεδάκι γιαουρτιού				
**Λαδερά** (π.χ. φασολάκια, αρακά,μπριάμ)	1,5 κεσεδάκι γιαουρτιού				
**Όσπρια** (π.χ. φακές, ρεβίθια - όχι φασολάκια, αρακά)	1 κεσεδάκι γιαουρτιού				
**Αυγά**	1 τεμάχιο				
**Κόκκινο κρέας** (π.χ. μοσχάρι, χοιρινό, αρνί, κατσίκι)	μέγεθος παλάμης				
**Λευκό κρέας** (π.χ. κοτόπουλο, γαλοπούλα, κουνέλι)	μέγεθος παλάμης				
**Αλλαντικά** (π.χ. γαλοπούλα, ζαμπόν, παριζάκι .κ.α.)	1 φέτα				
**Ψάρια & Θαλασσινά**	μικρά ψάρια (4-5 τεμάχια), μεγάλα ψάρια (1 τεμάχιο), θαλασσινά 0,5 πιάτο				
**Φρέσκες σαλάτες**	1 κεσεδάκι γιαουρτιού				
**Βραστές σαλάτες**	0,5 κεσεδάκι γιαουρτιού				
**Φρούτα** α) φρέσκα	1 μέτριο ή 2 μικρά φρούτα				
β) αποξηραμένα	3 τεμάχια για μεγάλα, π.χ. δαμάσκηνα ή 1,5 κουταλιά σούπας για μικρά (σταφίδες, κράνμπερις)				
**Γαλακτομικά προϊόντα**					
α) Πλήρη	γάλα (250 ml) ή 2 φέτες τυρί (40 γρ) ή τυρί κρέμα (120 γρ) ή τυρί φέτα (80 γρ) ή 1 κεσεδάκι γιαούρτι (200 γρ)				
β) Ημιαποβουτυρωμένα					
γ) Γάλα φυτικής προέλευσης (π.χ. αμυγδάλου, βρώμης, σόγιας)					
**Δημητριακά** (π.χ. κορν φλέικς, ή παρόμοια συσκευασμένα)	3 κ. σούπας				
**Γλυκά** α) <u>ΜΕ Ζάχαρη</u>	μπισκοτο 1τμχ, σοκολατα 1 μικρο τμχ, παγωτο 1 μικρή μπαλα, παστακι 1 τμχ				
β) <u>ΜΕ άλλα</u> γλυκαντικά (π.χ. στέβια)					

**Αλμυρά σνακ** (π.χ. γαριδάκια, πατατάκια, ποπ-κορν)	Απαντήστε πόσες φορές				
**"Γρήγορο φαγητό"** (π.χ. πίτσα, γύρος, κρέπα)	Απαντήστε πόσες φορές				
**Ξηροί καρποί**	1 χούφτα ξηρών καρπών , 1,5 κ σούπας βούτυρο καρπών				

2. Πόσα ποτήρια ή φλυτζάνια από τα παρακάτω ποτά καταναλώνετε κατά μεσό όρο;		σημειώστε αυτό που ταιριάζει περισσότερο			
	**Επεξήγηση μερίδας**	***Ποτέ / Σχεδόν ποτέ***	***Μερίδες /ημέρα***	***Μερίδες /εβδομάδα***	***Μερίδες /μήνα***
**Νερό**	<u>1</u> κούπα (250 ml)				
**Καφές** α) Με καφεΐνη	<u>0,5</u> κούπα (125 ml)				
β) Χωρίς καφεΐνη					
**Τσάι**	<u>0,5</u> κούπα (125 ml)				
**Χαμομήλι ή άλλα αφεψήματα**	<u>0,5</u> κούπα (125 ml)				
**ΣΥΝΟΛΙΚΑ <u>κουτ. γλυκού</u> ζάχαρης/μελιού στο ρόφημα την ημέρα;**	**Απαντήστε με έναν αριθμό:**	________			

		Ποτέ / Σχεδόν ποτέ	***Μερίδες /ημέρα***	***Μερίδες /εβδομάδα***	***Μερίδες /μήνα***
**Χυμός φρέσκος** α) Φρεσκοστιμμένος	<u>0,5</u> κούπα (125 ml)				
β) Συσκευασμένος	<u>0,5</u> κούπα (125 ml)				
**Αναψυκτικά** α) πλήρη σε θερμίδες	1 τενεκεδένιος περιέκτης (330 ml)				
β) χαμηλά σε θερμίδες (τύπου Light)					
**Αλκοόλ**	1 ποτήρι κρασιού (150 ml), 1 τενεκεδένιος περιέκτης (330 ml) μπύρας, 1 σφηνάκι βότκα, τζιν ουίσκι, ούζο (30 ml)				

		σημειώστε αυτό που ταιριάζει περισσότερο
3. Πώς επιλέγετε να μαγειρεύετε τα γευματά σας;		**Φορές /εβδομάδα**
**Τηγανητά**	πόσες φορές την εβδομάδα ετοιμάζετε κάτι με τον αντίστοιχο τρόπο;	
**Ψητά στον φούρνο/στη σχάρα**		
**Βραστά**		

4.Τι ποσότητα από τα παρακάτω είδη προστιθέμενων λιπών καταναλώνετε κατα μέσο όρο;		σημειώστε αυτό που ταιριάζει περισσότερο			
	**Επεξήγηση μερίδας**	***Ποτέ / Σχεδόν ποτέ***	***Μερίδες /ημέρα***	***Μερίδες /εβδομάδα***	***Μερίδες /μήνα***
**Ελαιόλαδο**	1 κουτ. σούπας				
**Φυτικά έλαια (καλαμποκέλαιο)**	1 κουτ. σούπας				
**Βούτυρο**	1 κουτ. γλυκού				
**Μαργαρίνη**	1 κουτ. γλυκού				
**Ελιές**	5 τεμάχια				
**Κρέμα γάλακτος** α) Χαμηλή σε λιπαρά	1 κουτ. γλυκού				
β) Κανονική					

	**Απαντήστε με έναν αριθμό**
5. Πόσα γεύματα <u>**συνολικά**</u> καταναλώνετε την ημέρα (κύρια γεύματα & σνακ);	
6. Πόσες ημέρες την εβδομάδα **συνήθως** κάνετε μεσημεριανό ύπνο **μετά** το φαγητό σας;	

Σας ενημερώνουμε πως **ΚΑΝΕΝΑ** στοιχείο που σας αφορά (προσωπικά στοιχεία, στοιχεία εγκυμοσύνης, συνήθειες διατροφής ή ό,τι άλλο) <u>**ΔΕΝ** θα κοινοποιηθεί σε τρίτους **ΟΥΤΕ** θα χρησιμοποιηθεί για άλλο σκοπό</u>, πέραν της ολοκλήρωσης της παρούσας μελέτης.
